# Design, characterization, and adsorption properties of *Padina gymnospora*/zeolite nanocomposite for Congo red dye removal from wastewater

**DOI:** 10.1038/s41598-021-00025-y

**Published:** 2021-10-26

**Authors:** Asmaa Ragab Dryaz, Mohamed Shaban, Hamad AlMohamadi, Khulood A. Abu Al-Ola, Ahmed Hamd, N. K. Soliman, Sayed A. Ahmed

**Affiliations:** 1 Department of Chemistry, Faculty of Science, Beni-Suef University, Beni Suef, 62511 Egypt; 2 Department of Physics, Faculty of Science, Islamic University in Almadinah Almonawara, Almadinah Almonawara, 42351 Saudi Arabia; 3 Nanophotonics and Applications Laboratory, Physics Department, Faculty of Science, Beni-Suef University, Beni Suef, 62514 Egypt; 4 Department of Chemical Engineering, Faculty of Engineering, Islamic University of Madinah, Madinah, Saudi Arabia; 5 Department of Chemistry, College of Science, Taibah University, Al-Madinah Al-Munawarah, 30002 Saudi Arabia; 6 Basic Science Department, Nahda University Beni-Suef (NUB), Beni Suef, Egypt

**Keywords:** Chemistry, Engineering, Materials science, Physics

## Abstract

A comprehensive study combined experimental, computational and field experiments was conducted to find out the most appropriate adsorbent system for industrial elimination of congo red (CR) dye from simulated industrial wastewater. Modification of the zeolite (Z) by the *Padina gymnospora* algae (PG) (Egyptian marine algae) was evaluated in terms of the adsorption capability of the zeolite (Z) to remove CR dye from aqueous solutions. The zeolite/algae composite (ZPG) was fabricated using the wet impregnation technique. Various techniques were used to characterize the PG, Z, and the produced ZPG nanocomposite. Batch experiments were performed to study the influence of various practical variables on adsorption processes. The isotherms and kinetics of dye adsorption were also studied. The newly synthesized ZPG nanocomposite exhibits much higher adsorption capacity, especially at low CR concentrations than that of Z. The computational calculations have shown that owing to the presence of intermolecular interactions, the adsorption of the CR molecule on zeolite surfaces is exothermic, energetically favorable, and spontaneous. For all configurations, increasing the zeolite size does not have a noticeable impact on the adsorption energies. The experimental results revealed that the ZPG nanocomposite can be applied as an economical nanoadsorbent to eliminate anionic dyes from simulated industrial wastewater at low CR dye concentrations. The adsorption isotherm of dye onto Z, PG, and ZPG almost agreed with Langmuir isotherm and pseudo-second-order kinetics. The sorption mechanism was also evaluated using Weber's intra-particle diffusion module. Finally, the field experiments revealed optimistic results for the newly synthesized adsorbent in removing dyes from industrial wastewater with 82.1% efficiency, which in turn confirmed the foundation of new eco-friendly materials that aid in the reuse of industrial wastewater.

## Introduction

Due to the continuous discharge of dyeing wastewater on fresh running water, the world is facing increasing pressure on water resources. Over 100,000 different dyes are currently available, with an annual production of more than 700,000 metric tons^1–3^. Even at low concentrations, the presence of dyes has obvious effects and toxicity on marine organisms by destroying the beautiful nature of water and reducing marine organisms' photosynthetic activity^4–6^. Among these dyes, Congo Red (CR) has received special attention due to its extensive use in the textile industry and its strong resistance to discoloration and biodegradation^7^. Therefore, it is critical to remove the dyes, particularly CR, from the wastewater before restoring it to the water system. Physical, chemical, and biological techniques are used for the removal of CR dyes. Adsorption, filtration, membrane separation, coagulation, dilution, flotation, softening electrochemical, and reverse osmosis are some of the techniques used^8–10^. Among these techniques, adsorption is the most practical for dye removal due to its low cost, ease of use, and low maintenance requirements, as well as the fact that the amount of sediment produced is less than that produced by the other technologies^11–15^. In the past few decades, biomass wastes from agricultural waste, algae, fly ash, and clay minerals have been used as effective and inexpensive absorbents for the removal of dyes and heavy metals from wastewater^4,16–21^. Because biomass wastes contain a variety of functional groups, including amino, carboxyl, carbonyl, hydroxyl, sulfonic acid, and phosphoric acid, these groups will induce pollutants bound to the walls of biological materials^22^. Zeolite is used not only in the field of water softening, but also in traditional applications of zeolite, and also in wastewater treatment, catalytic processes, antibacterial purposes, membrane separation, construction, coatings, pulp, and paper, plastics, refractories, and ceramics^23–26^.

In our work, a comprehensive study including computational, experimental, and field experiments is conducted to find out the most appropriate adsorbent system that effectively removes waste dyes especially CR dye from simulated industrial wastewater. The adsorption performance of Z, PG, and ZPG nanocomposite for CR dye removal from wastewater under different experimental conditions to investigate the influence of PG on the performance of Z as an adsorbent. However, such adsorbents are not novel at all, it has been firstly reported a long time ago and many times later. The innovation of this paper focused on the effect of the introduction of natural algae on zeolite adsorbent performance. PG and Z are nominated in this study because they are natural adsorbents of low cost. Furthermore, the cost of Z, PG, and ZPG regeneration for reuse is cheaper than that of many other methods, which could be a significant factor in its industry adoption. The effect of variables such as starting CR concentrations, nanoadsorbent doses, reaction periods, temperatures, and pH values on CR dye removal, adsorption kinetics, and isotherms was examined in batch mode experiments.

## Experiential details

### Raw materials, dyes, and reagents

Zeolite ore was supplied from El-Nassr company for mining and used as it is without further modification. The PG alga was collected from the intertidal zone of the Red Sea coasts between the cities of Marsa-Alam and Quoseir in Egypt. Sigma Aldrich provided the CR dye, which was dissolved in DI water. Sigma Aldrich provided 99.99% pure NaOH granules and 36% HCl, which were utilized to adjust the pH.

### Preparation of zeolite/algae (ZPG) composite

The zeolite/algae nanocomposite (ZUL) was fabricated using the wet impregnation technique. Zeolite/algae nanocomposite was prepared by the following steps, in the first step, 1 g of zeolite and 1 g of alga were mixed in 20 ml distilled water and stirred at 500 rpm for 60 min on a magnetic stirrer and then in ultrasonic for 60 min and repeated for 3 times and after that the resultant zeolite/algae nanocomposite was filtered and washed with distilled water for several times and dried in a vacuum oven at 60 °C for 24 h. The Z, PG, and ZPG nanocomposite were subjected to filtration, wash using DI water for many periods, and finally dried using a vacuum oven at 60 °C for 24 h. X-ray diffractometer (XRD), scanning electron microscope (SEM), Fourier transformer-infrared (FT-IR) spectrometer, and optical spectroscopy were used to characterize the Z, PG, and ZPG nanocomposite.

### Preparation of the adsorbate

CR is a well-known anionic dye, which used as the adsorbate in this experiment. CR's molecular formula is C_32_H_22_N_6_Na_2_O_6_S_2_, as shown in Figure S1 (Supplementary data). A 1000 mg/l stock solution was prepared by dissolving 1 g of CR dye in 1 l of DI water. The stock was then diluted with DI water to achieve the necessary working solution concentrations. Using either a 0.1 M HCl solution or NaOH solution, the pH of all prepared solutions was adjusted to 3, 5, 7, and 10.

### Samples characterizations

The PANalytical diffractometer (Empyrean) was utilized for the XRD measurements, which used a Cu(_Kα)_ source with a wavelength λα = 0.154045 nm and operated at 40 kV, 35 mA, with a scan step of 0.02° between 20° and 70°. The average crystallite sizes, D_s_, of the prepared nanoadsorbents were obtained by Scherer formula, D_s_ = 0.94 λα/β_w_ cosɸ; where β_w_ and ɸ are the corrected full width at half maximum and the diffraction angle^27^. Quanta FEG 250 microscope (Switzerland) was used to measure SEM micrographs. The dry KBr pellet method was used to measure FT-IR spectra by a Bruker VERTEX 70 FT-IR spectrophotometer.

### Adsorption studies

Four adsorption experiment series were implemented on Z, PG, and ZPG adsorbents at diverse adsorption circumstances, including initial dye concentration, adsorption temperature, adsorbent dosage, and initial pH of the solution, as displayed in Table S1 (Supplementary data). All CR adsorption experiments were done in batch mode scale under various experimental conditions including dye initial concentration (5–25 mg/l), contact time (up to 480 min), adsorbent dosage (0.01– 0.05 g per 20 ml of CR solution), pH (3–10), and temperature (25–90 °C) with continuous shaking.

The experiment time was set at 480 min and the volume of the solution was 20 ml in all experiments. The UV/Vis spectrophotometer was used to determine the variance in CR concentration by following the absorption peak. The reusability tests of Z, PG, and ZPG adsorbents were examined four times using 0.02 g of all adsorbents, 20 ml of 10 mg/l initial CR concentration for 480 min contact time at 25 °C and pH 7. Also, a reusability test was carried out for seven cycles using ZPG nanoadsorbent and 20 ml of 25 mg/l initial CR concentration. Z, PG, and ZPG adsorbents were collected from the solution after each run, then washed with DI water and set for the next run.

Equations (1) and (2) were used to calculate the amount of CR uptake by the synthesized nanocomposite at equilibrium (qe(mg/g) and time t (q_t_), as well as the CR dye removal%^28,29^.

1 $${\mathrm{q}}_{i}= \left({C}_{o}-{C}_{i}\right)\frac{V}{m};\mathrm{i }=\mathrm{ e},\mathrm{ t}$$

2 $$CR \, dye \, removal\mathrm{ \%}= \frac{({C}_{o}-{{C}_{t})}}{{C}_{o}} \times 100$$

At which C_o_, C_t_, and C_e_ are the concentrations of CR in mg/l at the start, after time t, and at equilibrium, respectively. V is the CR volume in mL and m is the Z, PG, and ZPG masses in mg. The presented results were the mean values of three independent experiments.

### Adsorption isotherm

Langmuir, Freundlich, and Tempkin isotherms have been applied to explain the reaction isotherm of the designed Z, PG, and ZPG nanocomposite for the tested CR^25^. All linear isotherms equations and their parameters are explained in supplementary data. The value of the dimensionless separation factor (R_L_) based on Eq. (3) could be used to predict the degree of favorability of the Langmuir isotherm for equilibrium data^30^.

3 $${\mathrm{R}}_{\mathrm{L}}=\frac{1}{(1+{\mathrm{K}}_{\mathrm{L}}{\mathrm{C}}_{\mathrm{max}})}$$

where C_max_ denotes the maximum initial CR concentration.

### Adsorption kinetics and mechanism

Different adsorption mechanisms and kinetics models such as intra-particle diffusion, pseudo-first-order, pseudo-second-order, and simple Elovich kinetic model are used for identifying the adsorption mechanisms and kinetics models that best match with the adsorption of CR onto Z, PG, and ZPG adsorbents^24,31–34^. All linear kinetics equations and their parameters are explained in supplementary data. The average values of all adsorption findings were measured in triplicates. The values of regression coefficients (R^2^) for various kinetic and isotherm models were obtained using OriginPro 2018's statistical functions.

### Computational calculations

The Zeolite Clinoptilolite and CR structures were optimized by density functional theory (DFT) using the GGA-PBE (Generalized Gradient Approximation-Perdew Burke Ernzerhof). The double numerical polarized (DNP) basis set was assigned. No spin-polarization effects were included in the exchange–correlation function. The core electrons of Zeolite Clinoptilolite and CR structures were treated with the effective core potential and all electrons, respectively. These calculations were carried out by the DMol3 module in the Biovia Materials Studio (version 2017, https://getintopc.com/softwares/simulators/biovia-materials-studio-2017-free-download/)^35–37^. The initial structure of Zeolite Clinoptilolite was obtained from this study^38^. The energy of the bulk structure of the zeolite unit cell was minimized, and then we built basic box nanoclusters with sizes of 3, 5, and 7 nm from the zeolite clinoptilolite using the nanocluster builder implicit in Materials Studio software, as follows: simple box, 3 nm (X,Y, Z directions = 30 Å, K = 64, Na = 132, Si = 468, O = 1220, Ca = 56, Al = 522, H = 224 atoms), 5 nm (X,Y, Z directions = 50 Å, K = 249, Na = 492, Si = 2213, O = 5520, Ca = 270, Al = 1342, H = 1044 atoms), and 7 nm (X,Y, Z directions = 70 Å, K = 684, Na = 6112, Si = 468, O = 8834, Ca = 644, Al = 3214, H = 2720 atoms). Using Monte Carlo (MC) simulation, we investigated the effect of different sizes of Zeolite Clinoptilolite on the adsorption energy and found the desorption sites of CR on the Zeolite Clinoptilolite surface. The adsorption locator module in materials studio was used to do the MC simulation, which used the COMPASS force field (Condensed-phase Optimized Molecular Potentials for Atomistic Simulation Studies) as a force field and current in the charges section. The basic principles of MC simulation have been described by Frenkel and Smit^39^

### Field experiments

The newly synthesized composite was tested as an effective eco-friendly adsorbent that could be used to remove industrial waste dyes from industrial wastewater on a large scale. Wastewater containing waste dye was supplied by clothes dying plant in Beni-Suef city for this purpose, and the wastewater containing waste dye was used untreated and undiluted. The optimum catalytic system was chosen based on our reorganized computational and experimental results.

## Results and discussion

### Adsorbent characterizations

#### SEM characterization

**Figure 1:**
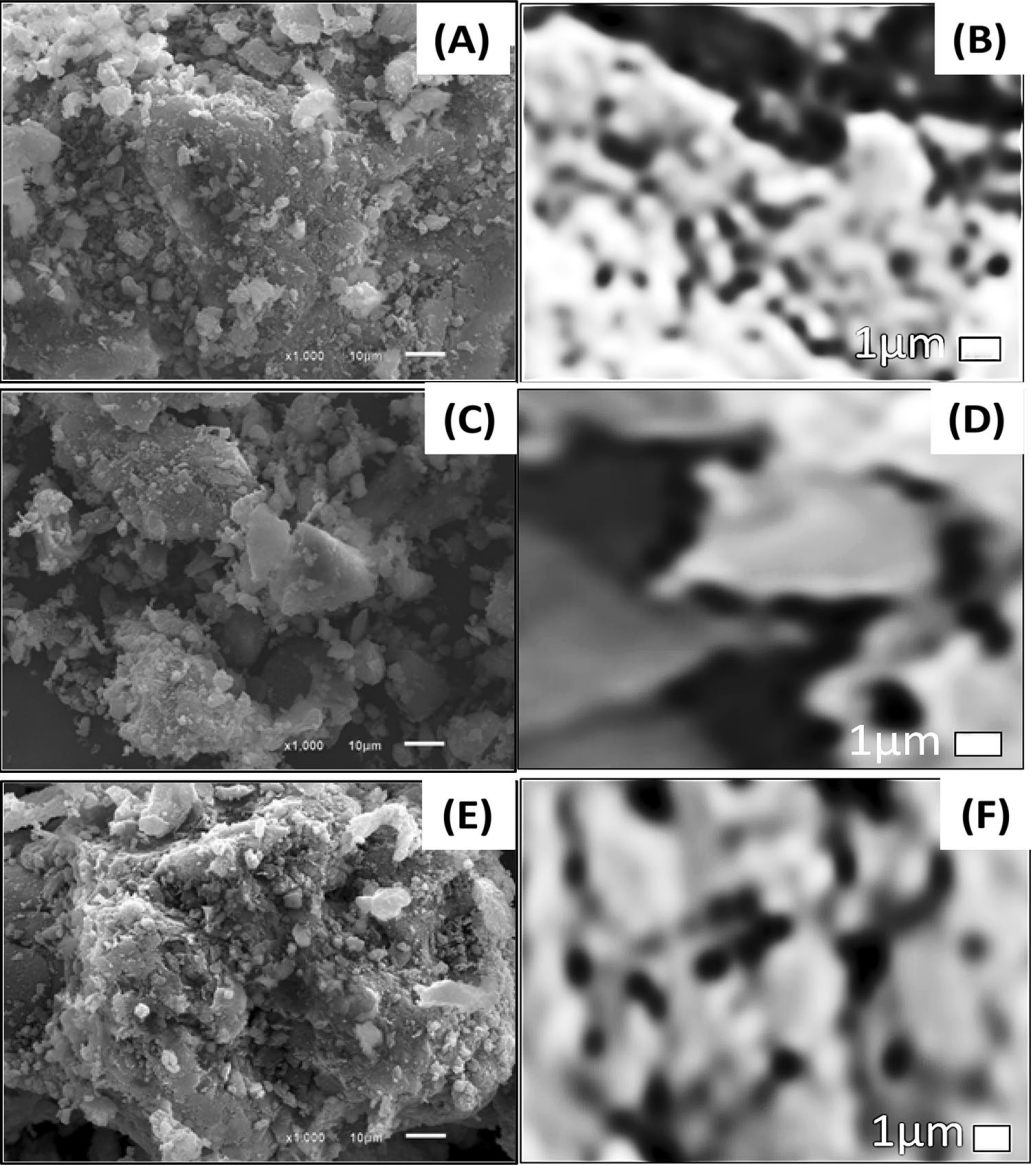
SEM micrographs of (**A**,**B**) Z, (**C**,**D**) PG, and (**E**,**F**) ZPG adsorbents.

The SEM images of Z, PG, and ZPG adsorbent are illustrated in Fig. 1. Figure 1A,B shows SEM images of zeolite with agglomerated rounded regular shape particles, rough surface, different particle sizes, and porous cavities on the surface. The average diameter of the pores is 514 ± 89 nm. Figure 1C,D displays SEM images of PG with a less porous surface which consequently affects the surface area for PG which in turn affects its adsorption capacity. The density of pores on the PG surface is lower than on the Z surface, but the diameter of the detected pores on the PG surface (849 ± 134 nm) is greater than on the Z surface. When zeolite is treated with PG algae, Fig. 1E,F, the SEM image of the nanocomposite shows that the pores in the zeolite surfaces are covered with agglomerated PG particles. Because nearly all small pores are sealed, we only found broad pores with an average diameter of 817 ± 120 nm. The formation of ZPG nanocomposite could be established from alterations in the nanocomposite's morphological topographies when compared to those of Z and PG. The DLS analysis is used to calculate the hydrodynamic diameter and particle size distribution of the Z, PG, and ZPG particles (Supplementary data, Figure S2). Z, PG, and ZPG have average hydrodynamic diameters of 80.6, 95.0, and 91.4 nm, respectively. Also, the PET surface areas for Z, PG, and ZPG are measured to be 91.2, 126.7, and 117.3 m^2^/g, respectively.

#### XRD characterization

**Figure 2:**
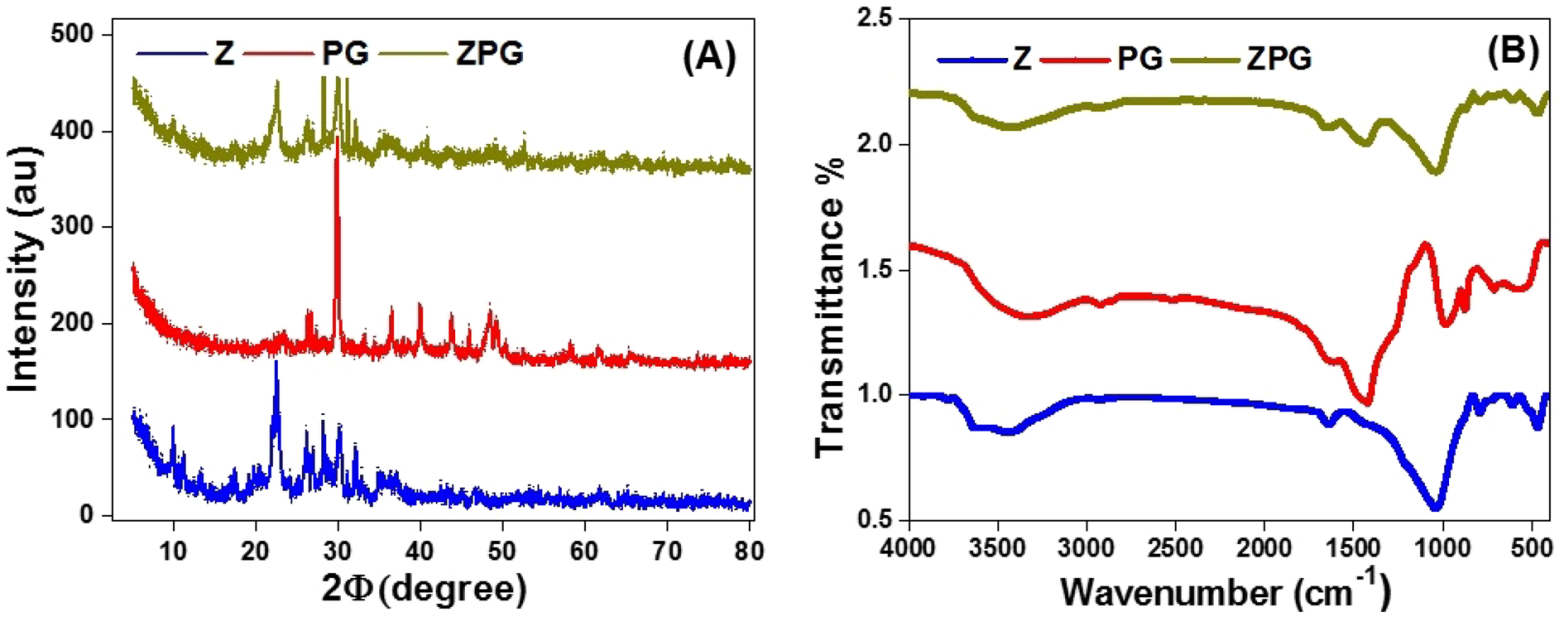
(**A**) XRD and (**B**) FTIR charts of Z, PG, and ZPG adsorbents.

Figure 2A illustrates XRD charts of Z, PG, and ZPG adsorbents. The main XRD peaks of zeolite minerals appear at 2ɸ of ~ 9.85°, 22.41°, 26.15°, 26.84°, 28.12°, 30.075°, and 32.04° which are in good agreement with those reported by other researchers^40,41^. The obtained d-spacing values of the main peaks of zeolite at 22.41° and 28.12° are 3.96809 Å and 3.17323 Å. The main XRD peaks of PG appear at about 26.23, 26.61°, 29.77°, 39.87°, and 48.38°, while the pattern of ZPG shows characteristic main peaks at about 22.49°, 26.15°, 28.16°, 29.96°, 31.09°, 32.07° and 40.73°. The average crystallite size using the Scherrer equation was ~ 45 nm, which confirms the nanoscale nature of the newly synthesized composite.

#### FT-IR analyses

FT-IR charts of Z, PG, and ZPG adsorbents are shown in Fig. 2B. The broad bands in the FT-IR spectrum which displayed from 3320 to 3420 cm^−1^ refer to the stretching hydroxyl (OH) groups^42–44^. The band at 1029 cm^−1^ is associated with Si–O vibration mode in the case of zeolite, which is shifted to 1039 cm^−1^ for ZPG^45^. The bands at 603 and 1029 cm^−1^ are related to the Si–O-Al, and octahedral aluminum (Al–OH)^46^. The band at 464 cm^−1^ is related to the Si–O-Si bending of zeolite, which is shifted to 461 cm^−1^ for ZPG^46^. The bands that appeared in the region from 400 to 800 cm^−1^ are related to the metal oxides^47^.

The FT-IR spectrum of PG alga shows bands at 3624 and 3432 cm^−1^ related to the amine group (-NH) stretching and the hydroxyl group (–OH) of phenolic groups. The band at 2935 cm^−1^ is allocated to the alkyl (CH) group stretching mode, whereas the band at 1637 cm^−1^ is due to O–H vibration (from binding water or –OH). The band at 1425 cm^−1^ is attributed to the C–H mode^48^. The bands located around 1019 cm^−1^ refer to the sulfate group or the C–O bond^49^. While modes around 3300 – 3500 cm^−1^ are characteristic of the N–H stretching mode of amines. The bands around 2925 and 2935 cm^−1^ refers to the vibration of saturated C–H. Both the band shift and band disappearance come in line with data obtained from other characterization techniques which confirms the formation of a new compound. Table S2, supplementary data, lists the positions of the characteristic FT-IR bands for Z, PG, and ZPG adsorbents.

### Factors influencing the adsorption process

#### Influence of initial CR concentration

**Figure 3:**
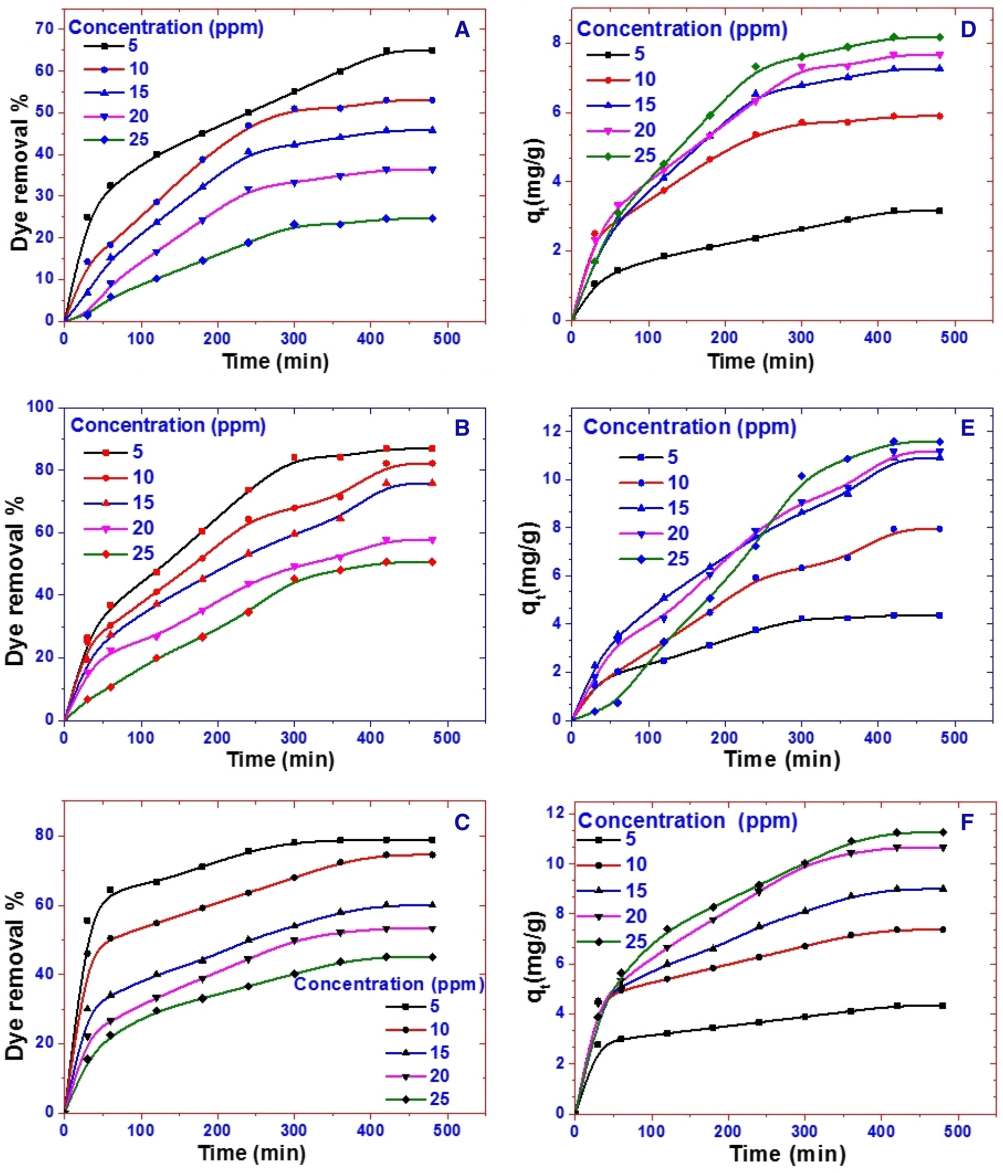
Effect of CR dye concentration and contact time on the removal% and the amount of CR dye adsorbed at 25 °C and pH 7 by 20 mg of (**A**,**D**) Z, (**B**,**E**) PG, and (**C**,**F**) ZPG.

The amount of dye removed by adsorption is heavily influenced by the adsorbate's starting concentration. The variations in the removal % and the amount of CR adsorbed with time using Z, PG, and ZPG nanoadsorbents at different initial CR concentrations are shown in Fig. 3A–C; D–F, respectively. During the first stage of the adsorption process, the amount of dye removed and the dye removal percentage were normally very high, and then steadily reduced until they achieved equilibrium. After reaching equilibrium, contact time has no discernible effect on the adsorption process using new sorbents.

The rapid removal rate at the early stage in the adsorption progression is due to the presence of a large number of uncovered active adsorption spots on the adsorbent's surfaces. The hot spots were converted to fully occupied sites by CR molecules through rising the contact period between adsorbent and adsorbate. As a result, repulsion forces are increased between CR molecules adsorbed on adsorbent surfaces and CR molecules in the bulk liquid phase^29^. By increasing the initial CR dye concentration, the dye removal percentage decreases. In contrast, the high driving force for mass transfer at an elevated starting CR concentration is the main reason for the rising of the adsorbed CR quantity by the adsorbent.

The clay nanocomposite, ZPG, revealed higher efficiency than Z for CR adsorption at all concentrations and the CR removal% was in the order PG > ZPG > Z. As shown in Fig. 3D–F, the quantities of CR adsorption are increased by increasing the starting CR concentration. This could be accredited to the growth of the concentration gradient with rising the starting CR concentration. Hence, appropriate growth in the draft forces occurs to overawed the mass transfer resistance between the CR adsorbate and Z, PG, and ZPG adsorbents^50,51^. The maximum adsorption capacities of ZPG were found to be 4.3, 7.4, 9.0, 10.7, and 11.3 mg/g for CR dye solutions of concentrations 5, 10, 15, 20, and 25 mg/l, respectively, at pH 7 and 25 °C. In the same order, the adsorption capacities of PG were found to be 4.4, 8.0, 10.9, 11.2, and 11.6 mg/g. Additionally, the maximum adsorption capacities of Z were found to be 3.2, 5.9, 7.3, 7.7, and 8.2 mg/g at these starting concentrations. The results showed that the modification of Z with PG is a feasible approach to enhance the CR removal performance by Z at all concentrations.

#### Influence of nanoadsorbent dose

**Figure 4:**
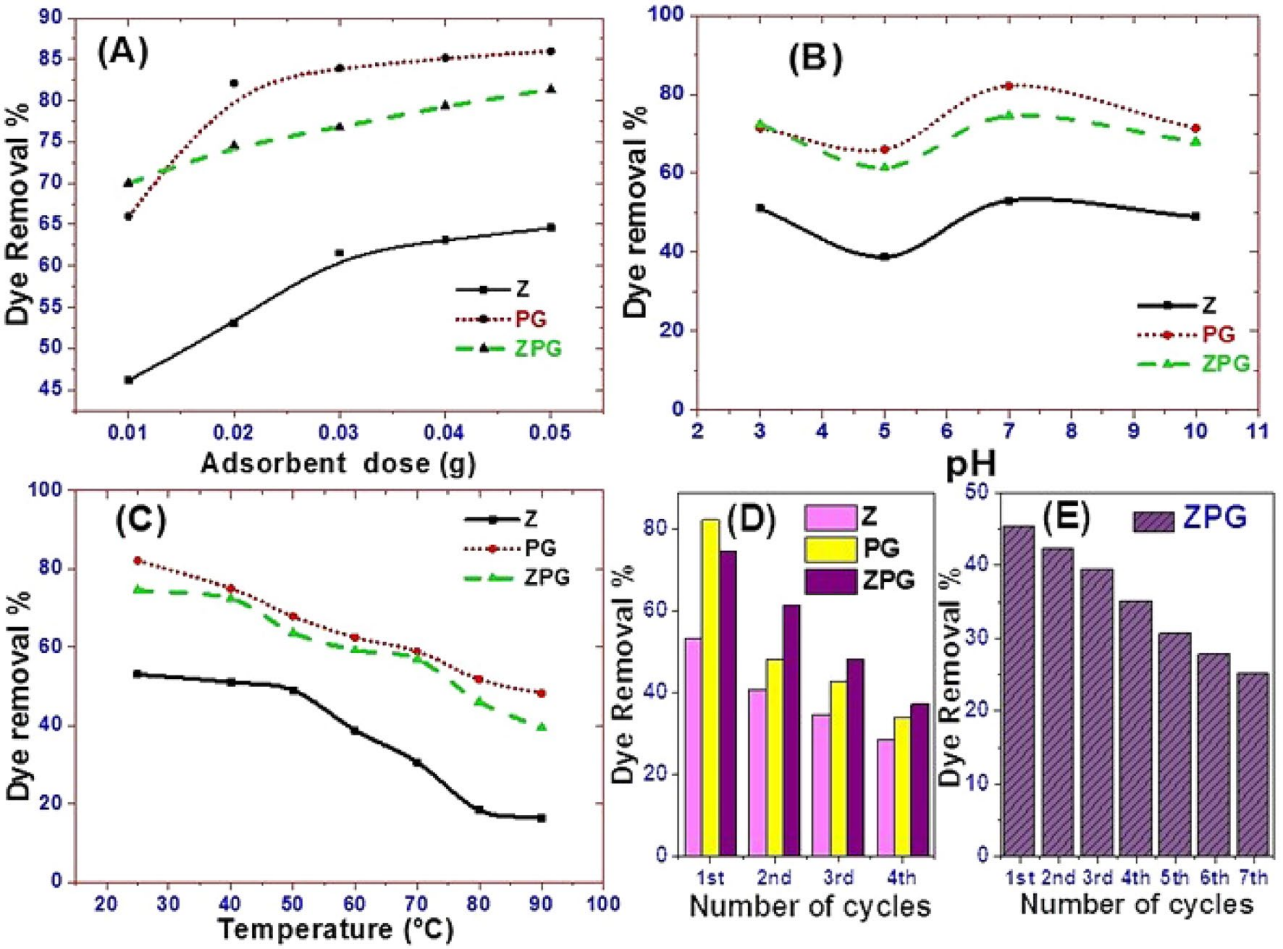
Effect of (**A**) adsorbent weight, (**B**) initial pH of the solution, (**C**) adsorption temperature, and reusability test on the removal% of 20 ml CR solution of (**D**) 10 mg/l by Z, PG, and ZPG and (**E**) 25 mg/l by ZPG.

The impact of the nanoadsorbent dose on the CR removal percent was evaluated to determine the optimal nanoadsorbent dosage that provides the best performance to determine the adsorption cost. Figure 4A illustrates graphically the change in the CR dye removal% as a function of adsorbent dosage. The adsorbent doses were varied from 0.01 to 0.05 g. The CR removal% is increased as the adsorbent dose raised from 0.01 to 0.05 g for Z, PG, and ZPG adsorbents. The removal% increases from 46.15 to 64.62% in the case of Z adsorbent, from 66.00 to 86.00% in the case of PG adsorbent, and from 70.00 to 81.36% in the case of ZPG adsorbent. This observation could be ascribed to the increase of the number of hot spots with increasing the nanoadsorbent dosage^31,51,52^. It was observed that a large jump in removal% takes place by increasing the nanoadsorbent dose from 0.01 to 0.02 g for ZPG, and from 0.01 to 0.03 g for Z. The change in removal percent became slightly smaller when the adsorbent dose was increased above 0.02 g and 0.03 g in the case of ZPG and Z, respectively. A dense screening layer arises at the nanoadsorbent surfaces when adsorbent molecules accumulate and the distance between adsorbent molecules decreases. When the dosage of nanoadsorbents is raised, the "screening effect" occurs. As a result, the condensed layer on the adsorbent's surface obscured the binding sites from CR molecules. Furthermore, because Z and ZPG overlapped, CR molecules competed for a limited number of accessible binding sites. Agglomeration or aggregation at higher Z and ZPG dosages lengthens the diffusion channel for CR adsorption, lowering the adsorption rates^53–55^.

#### Influence of pH

The pH value is a crucial player in controlling the nanoadsorbent performance for wastewater treatment. The variation in pH leads to an alteration in the ionization degree of the adsorptive molecules and the adsorbent surface properties. Figure 4B shows the impact of pH value on the CR elimination% by the adsorbent. The pH effect was studied between pH 3 and pH 10 at an initial CR concentration of 10 mg/l and sorbent dosage of 0.02 g**.** The Z adsorbent shows removal percentages of ~ 51%, 38%, 53%, and 49% for CR solutions of pH 2, 5, 7, and 10; in that order. The PG adsorbent shows removal percentages of ~ 71.4%, 66%, 82.1%, and 71.4%, while ZPG adsorbent shows removal percentages of 72.3%, 61.4%, 74.5%, and 67.9% at pH 2, 5, 7, and 10, respectively, at the same previously mentioned conditions. As shown in Fig. 4B, when the pH is 7, the adsorption capacity of CR on Z, PG, and ZPG reached its maximum. This may have resulted from that the interaction between Z, PG, and ZPG and CR is more pronounced than the interaction with OH^−^ ions on the solution^24^.

For CR dye solutions with low pH values, the positive charge on the solution/adsorbent interface will increase and the Z, PG, and ZPG surfaces will appear to be positively charged. However, CR in the solution tends to be neutral due to protonation. This condition leads to a reduction in the anionic CR dye adsorption at pH 5^56^. At high pH, the decrease in the positive charge on the solution interface takes place and CR dye molecules will become negatively charged with OH^−^ ions. Therefore, positively charged Z, PG, and ZPG have a competitive interaction with negatively charged OH^−^ ions or CR dyes. Consequently, when pH > 7, CR dye removal% will decrease again^57^.

#### Influence of temperature

The influence of the temperature on CR dye adsorption is an additional considerable Physico-chemical processing factor since it will cause variation in the adsorption capability of the adsorbent^58^. The adsorption tests were done at 25, 40, 50, 60, 70, 80, and 90 °C as shown in Fig. 4C. A decrease in CR removal% is observed with increasing temperature. This may be allocated to the desorption behaviors that resulted from the destruction of adsorption forces between hot binding spots of the nanoadsorbent and the CR adsorbate species, which may be due to the destruction of active sites^51,59–61^. Therefore, the optimum temperature for adsorption of CR onto all tested adsorbents is 25 °C. The decrease in CR removal percentage with increasing temperature indicates that the adsorption process is exothermic.

#### Reusability test

A biosorption/desorption reusability test can be carried out to determine the most effective eluent for dye desorption and to investigate the bio sorbents' reusability. Nitric acid (HNO_3_), distilled water, or deionized water can all be used as eluents. However, because high nitric acid concentrations can damage biosorbent structure, further research is needed to address this issue utilizing different eluents with low concentrations to avoid possible harm to biosorbent structure. As a result, using distilled water in the adsorbent regeneration is reasonable and will minimize the cost of the treatment process because distilled water is a low-cost eluent. Figure 4D shows that the Z, PG, and ZPG reusability tests for the elimination of CR were performed four times with the same adsorbent and dose. The removal strength of all used adsorbents varied considerably throughout the four adsorption cycles, according to the findings. For Z adsorbent, the recorded dye removal% was 53.06%@1st cycle, 40.82%@2nd cycle, 34.69%@3rd cycle, and 28.57%@4th cycle. For PG adsorbent, the dye removal% was decreased from 82.14% @1st cycle to 33.92% @4th cycle. For ZPG nanoadsorbent, the dye removal% was decreased from 74.56%@1st cycle to 37.28% @4th cycle. The reusability of ZPG is also tested for seven cycles using 20 ml CR solutions of concentration 25 mg/l. The dye removal% was decreased from 45.27% @1st cycle to 25.09% @7th cycle. The drop in the CR removal% could be ascribed to the agglomeration of the CR molecules onto the surface of Z, PG, and ZPG adsorbents, which consequently hind the adsorbent surface and pores from the dissolved CR molecules and so, a reduction in adsorption capacity take place^62^.

### Adsorption isotherm

**Figure 5:**
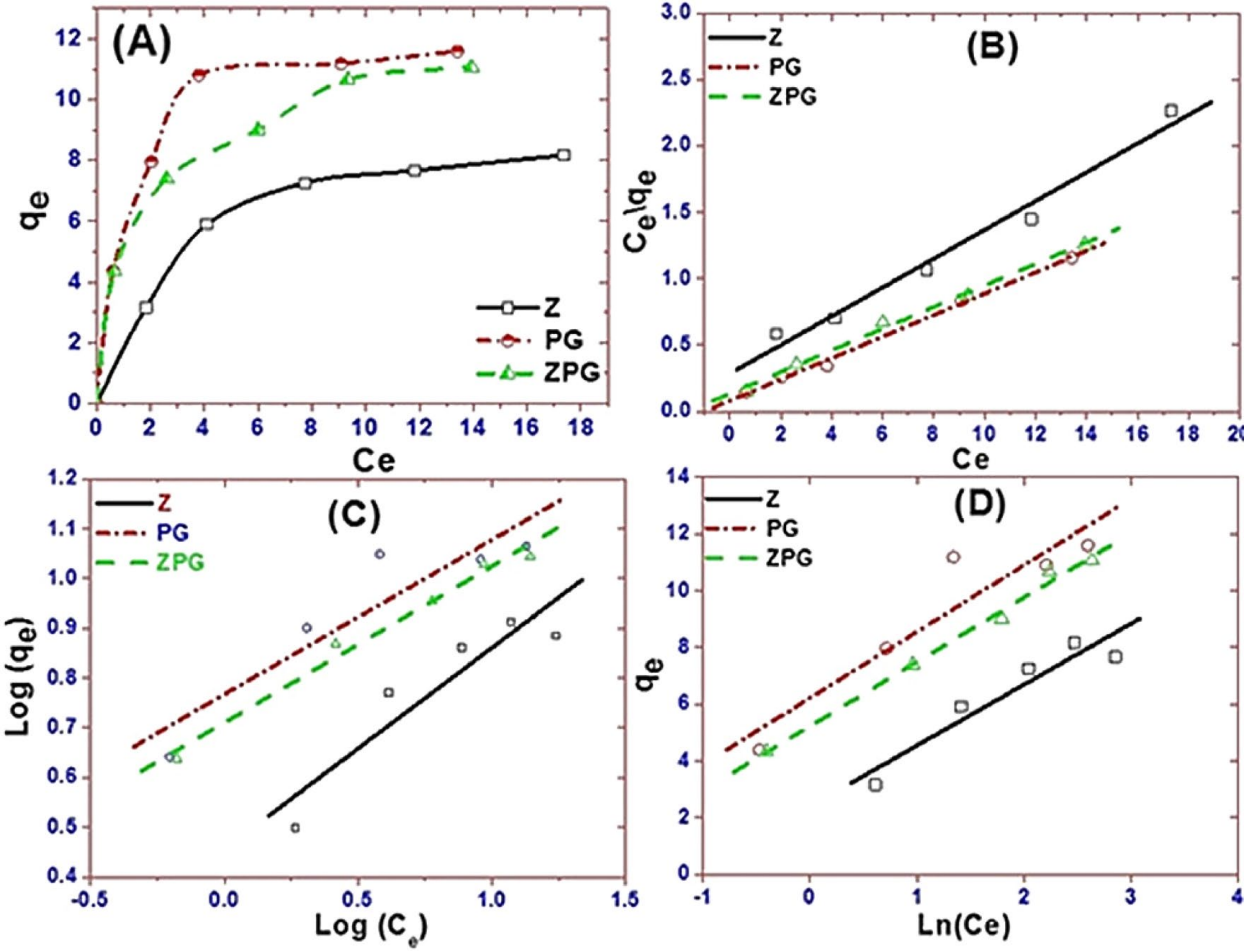
Plots of (**A**) qe vs. Ce, (**B**) Langmuir, (**C**) Freundlich, and (**D**) Temkin adsorption isotherms for the adsorption of CR dye by 20 mg of Z, PG, and ZPG at 25 °C and initial pH 7.

Curves of q_e_ vs. C_e_ for Z, PG, and ZPG adsorbents are presented in Fig. 5A. These isotherm curves directly show the property of equilibrium adsorption. The statistical significance of R^2^ (correlation coefficient) for the linear fitting of Ce/qe versus Ce, log(qe) versus log(Ce), and qe versus ln(Ce) was used to fit the data to the Langmuir, Freundlich, and Tempkin isotherms, individually. From the linear plots, Fig. 5B–D, the values of K_L_, K_F_, K_T_, Q_o_, n, B, and R^2^ were determined and recorded in Table 1. The results in Table 1 demonstrate that CR adsorption on Z, PG, and ZPG adsorbents tracks the Langmuir isotherm models where the R^2^ value is the highest. I.e., the adsorption process almost tracks the Langmuir isotherm model. As a result, the elimination of the dye occurs at the active sites of the nanoadsorbents on a single surface layer, and the adsorbed CR molecules do not interact with each other. At 25 °C, the obtained R^2^ values by the Langmuir isotherms of Z, PG, and ZPG adsorbents were 0.979, 0.996 and 0.995, respectively. The value of R_L_ is < 1, indicating that the adsorption of CR is favorable in the study case^63^. The maximum amount of CR adsorbed on the surface of Z, PG, and ZPG according to the Langmuir isotherm model was expected to be 9.23, 12.38, and 12.25 mg/g.

**Table 1:** Isotherm parameters for CR adsorption on Z, PG, and ZPG.

Constant	Langmuir isotherm			
	Q_o_(mg/g)	K_L_(L/mg)	R _L_	R^2^
**Adsorbent**				
ZPG	12.25	0.62	0.06	0.995
PG	12.38	1.03	0.03	0.996
Z	9.23	0.38	0.09	0.979

Constant	Freundlich isotherm			
	1/n	K_f_	R^2^	
**Adsorbent**				
ZPG	0.31	5.12	0.982	
PG	0.31	5.83	0.850	
Z	0.40	2.85	0.856	

Constant	Temkin isotherm			
	B(J/mol)	K_T_(L/mole)	R^2^	
**Adsorbent**				
ZPG	2.26	10.00	0.992	
PG	2.34	14.21	0.876	
Z	2.15	3.04	0.896	

### Adsorption kinetic models

**Figure 6:**
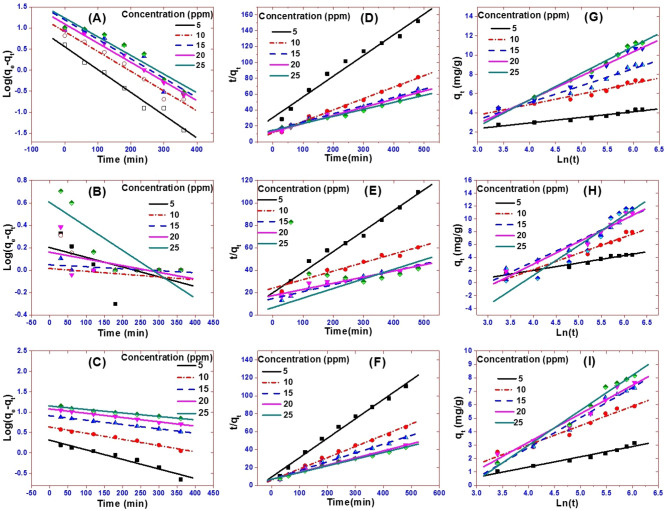
(**A**–**C**) Pseudo-first-order, (**D**–**F**) Pseudo-second-order, and (**G**–**I**) Elovich sorption kinetics of CR dye at 25 °C and pH 7 by 20 mg of Z, PG, and ZPG, respectively.

To investigate the most appropriate adsorption kinetics model, the adsorption of CR on Z, PG, and ZPG under various starting CR concentrations was addressed. The first-order, second-order, and Elovich kinetics linear graphs were represented in Fig. 6 by plotting ln(q_e_ − q_t_) vs. t, $\frac{\mathrm{t}}{{\mathrm{q}}_{\mathrm{t}}}$ vs. t, and q_t_ vs. ln(t), respectively. The adsorption kinetics parameters k_1_, k_2_, q_e_, β, and α of the evaluation model in addition to R^2^ were obtained using the linear plots and depicted in Table 2.

**Table 2:** Parameters of the kinetic models for CR dye adsorption on Z, PG, and ZPG.

Adsorbent	Conc, ppm	First-order				Second-order				Elovich kinetic model		
		q_e exp_	q_e calc_	k_1_	R^2^	q_e_ exp	q_e calc_	k_2_	R^2^	β (g/mg)	Α(mg/min)	R^2^
Z	5	3.15	3.58	0.012	0.9800	3.15	3.82	0.0020	0.9752	1.310	0.080	0.9597
	10	5.89	8.15	0.0108	0.9694	5.89	6.92	0.0017	0.9918	0.700	0.210	0.9575
	15	7.26	15.6	0.0106	0.8805	7.26	9.67	0.0007	0.9918	0.450	0.140	0.9835
	20	8.17	12.4	0.0105	0.9820	8.17	9.72	0.00085	0.9855	0.470	0.180	0.9630
	25	7.67	17.2	0.0102	0.9163	7.67	11.31	0.00055	0.9891	0.380	0.150	0.9835
PG	5	4.38	1.59	0.002	0.2930	4.375	4.52	0.002484	0.9837	0.863	0.115	0.9621
	10	7.96	1.03	0.0006	0.0096	7.9591	13.11	0.000244	0.9476	0.396	0.106	0.9471
	15	11.19	1.12	0.00041	0.3060	11.186	16.16	0.000262	0.9485	0.304	0.165	0.9434
	20	10.91	1.44	0.00137	0.2436	10.91	18.19	0.000175	0.9349	0.286	0.148	0.9443
	25	11.59	4.01	0.0049	0.7177	11.594	11.68	0.001132	0.0944	0.214	0.110	0.9198
ZPG	5	4.33	2.03	0.0054	0.9435	4.33	4.62	0.005034	0.9950	1.709	1.636	0.9289
	10	7.37	4.3	0.0034	0.9806	7.37	7.96	0.002727	0.9911	0.910	1.681	0.9446
	15	9.00	8.1	0.0024	0.9968	9	10.16	0.001436	0.9868	0.566	0.567	0.9551
	20	10.67	11.88	0.0024	0.9960	10.67	12.61	0.000923	0.9875	0.401	0.388	0.9665
	25	11.27	14.07	0.00196	0.9796	11.27	13.37	0.000818	0.9925	0.362	0.349	0.9912

The linear fit and regression coefficient values ​​in Table 2 for all the studied kinetic models confirmed that CR adsorption onto Z, PG, and ZPG is well handled with the second-order model at all CR concentrations. This was also confirmed from the good approximation between the calculated qe and experimental q_exp_. The pseudo-second-order adsorption mechanism is performed in two stages. The first stage is an external diffusion stage, which includes the movement of CR molecules from all sides to the outer surfaces of Z and PG. This is followed by a second stage in which CR molecules adsorb and bind to the surfaces of Z, PG, and ZPG.

### Sorption mechanism

The practical data is fitted for Weber's intra-particle diffusion to better understand the adsorption kinetics process and rate-controlling steps. The applicability of the intra-particle diffusion model is suggested by the linear plot of q_t_ versus t^1/2^, Figure S3 (Supplementary data). The slope and intercept of the linear fitting of Figure S3 were used to calculate the values of the intra-particle propagation model rate constant (K_3_) and boundary thickness constant (I) in Table 3. The value of intercept I is not zero, demonstrating that the intra-particle diffusion model may not be the sole rate-controlling factor in determining the kinetics of the adsorption process^64^. The intercept in Figure S3 refers to the boundary layer effect. The larger intercept, the greater the contribution of surface adsorption in the rate-controlling stage^64^.

**Table 3:** Intra-particle diffusion constants for different initial CR concentrations at 25 °C.

Adsorbent	Concentration (ppm)	Intraparticle diffusion kinetic model		
		I	k_3_ ( mg/g min ^1/2^)	R^2^
**Z**	5	0.1670	0.1456	0.9907
	10	0.5095	0.2931	0.9661
	15	− 0.0329	0.38143	0.9822
	20	0.1675	0.3886	0.9899
	25	− 0.1748	0.4348	0.9806
**PG**	5	0.5482	0.1911	0.9610
	10	− 1.1082	0.4256	0.9876
	15	− 0.8973	0.5570	0.9919
	20	− 1.5232	0.5892	0.9860
	25	− 4.8178	0.7909	0.9707
**ZPG**	5	2.1842	0.0996	0.9881
	10	3.4590	0.1855	0.9892
	15	2.8271	0.2964	0.9891
	20	2.2558	0.4142	0.9790
	25	2.0124	0.4531	0.9787

### Computational analysis

#### MC simulation

**Figure 7:**
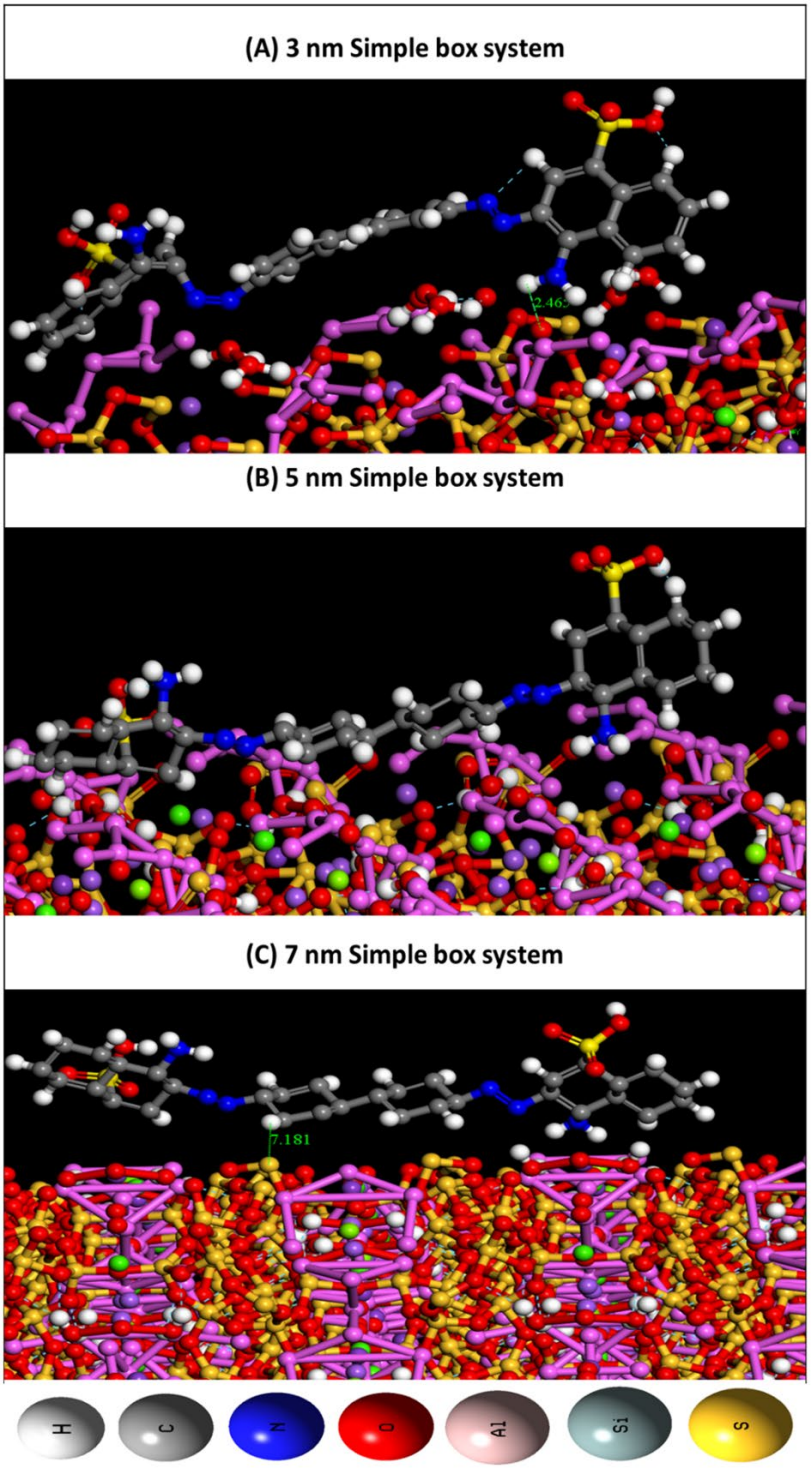
Snapshots for the adsorption configurations of CR/Zeolite Clinoptilolite of 3, 5, and 7 nm simple box systems, the bond length is in Angstroms.

The lowest configurations obtained due to the adsorption of CR on zeolite clinoptilolite for different three sizes are summarized in Fig. 7. MC simulation aims to elucidate the influence of diverse planes and sizes of zeolite clinoptilolite on the adsorption of CR. The adsorption energies of each zeolite clinoptilolite-CR system are titled in Table 4. The complete simple box systems for adsorption configurations of CR on zeolite clinoptilolite are displayed in Figure S4 (Supplementary data) for clarity purposes. Figure 7A–C shows the snapshots of CR adsorption on the zeolite clinoptilolite of 3, 5, and 7 nm simple box systems in a dry system without any solvent. The CR molecule holds various hydrogen bond (HB) donor/acceptor spots, and hence, it forms a number of HBs with the nitrogen and oxygen atoms of the zeolite clinoptilolite. The oxygen and the nitrogen atoms of CR form HBs and intramolecular HBs through the hydroxyl hydrogen atoms of the zeolite clinoptilolite.

**Table 4:** Adsorption energies for the adsorption configurations of Congo red adsorbed on Zeolite Clinoptilolite with 3, 5, and 7 nm simple box systems.

System	Adsorption energy	Rigid adsorption energy	Deformation energy	Congo red : dE_ad_/dN_i_
3 nm	− 42.22350801	− 32.58960227	− 9.63390575	− 42.22350801
5 nm	− 40.83882700	− 29.74763343	− 11.09119357	− 40.83882700
7 nm	− 38.15356471	− 28.49577995	− 16.65778476	− 38.15356471

Figure 7A–C displayed the formation of HBs and intramolecular HBs between hydrogen atoms of CR and the atoms of the zeolite clinoptilolite with 5 and 7 nm simple box systems. Table 4 shows the adsorption energy, E_ads_, the interaction energy, E_int_, the deformation energy (E_def_), and the substrate/adsorbate configurations (dE_ads_/dN_i_), in which one of the adsorbate constituents is missing. In this study, the ΔE_ads_ for all configurations is negative, which agrees with the experimental results. I.e., the adsorption reactions of CR molecules on zeolite clinoptilolite with different sizes are exothermic, energetically favored, and spontaneous because of the intermolecular interactions. Furthermore, increasing the zeolite clinoptilolite size has a major impact on the adsorption energies for all configurations. The adsorption energies decrease as the size of the simple box increases. In the 5 and 7 nm simple boxes, HBs and intramolecular HBs between CR and the zeolite clinoptilolite are weaker than in the 3 nm simple box. This causes a decrease in ΔE_ads_ value, whereas the adsorption energies for all configurations in the state of 3 nm simple box system are larger than those in 5 and 7 nm simple box systems, as shown in Table 4.

### Field experiments and comparison with previously reported adsorbents

The newly synthesized ZPG adsorbent's optimal parameters were 0.02 g adsorbent mass, near room temperature, and 420 min contact time, while the pH of the wastewater containing the waste dye remained unchanged. Optical scanning of the as-received industrial wastewater revealed the presence of different wavelengths corresponding to different dyes. At the end of the contact period, absorbance at different wavelengths was recorded to determine the dye removal% from industrial wastewater. Field studies showed promising results for the newly synthesized adsorbent in removing dyes from industrial wastewater with 82.1% efficiency, confirming the foundation of new eco-friendly materials that aid in the reuse of industrial wastewater.

Table 5 compares the optimized conditions, removal percent, and adsorption capacity, q_m_, of our investigated Z, PG, and ZPG nanoadsorbents for CR dye removal to those previously reported in the literature for multiple adsorbents^65–69^. This table shows that our optimized ZPG nanoadsorbent outperformed the majority of previously reported CR adsorbents^65–69^.

**Table 5:** Comparison of the optimized conditions, removal%, and adsorption capacity of different CR adsorbents relative to our Z, PG, and ZPG nanoadsorbents.

Adsorbent	Conditions	Adsorption capacity (mg/g)	Removal %	References
Green Coffee Residue (GCR)	Adsorbent Dose: 40 mg/L Concentration: 100 mg/l Time: 800 min pH: 6 Temperature: 25 °C	20.04	99.04%	^65^
CTAB functionalized walnut shell	Adsorbent Dosage: 1 g/L Concentration: 100 mg/l Time: 360 min pH: 6 Temperature:29 °C	104.4	–	^66^
cashew nut shell	Adsorbent Dosage: 20 g/L Concentration: 20 mg/l Time: 120 min pH: 3 Temperature: 30 °C	5.18	98.52%	^67^
Typhaaustralis Leaves	Adsorbent Dose: 0.2 g/L Concentration: 10 mg/l Time: 60 min	24.23 at 40 °C 21.85 at 30 °C 17.40 at 20 °C	–	^68^
Water hyacinth roots	Adsorbent Dosage:1 g/L Time: 180 min pH: 6 Temperature: 25 °C	13.46	46.15	^69^
ZPG	Contact time: 480 min Adsorbent dose: 0.02 g Concentration: 20 mg/L pH: 7.0 Temp: 25 °C	11.26	78.89%	This work
PG		11.59	86.84%	
Z		8.1	65%	

## Conclusion

The hydrothermal treatment of Z and PG resulted in the formation of a novel alga/zeolite (ZPG) nanocomposite, which was employed as a new adsorbent for CR from aqueous solutions. The morphologies and structures of Z, PG, and ZPG have been investigated, and it has been observed that PG nanoparticles and Z nanopores aggregate to form 45 nm nanocomposite crystallites. The experimental results revealed that the CR removal% generally improved with reducing the starting CR concentration and reaction temperature. The removal% increased by increasing the adsorbent dosage from 0.01  to 0.05 g to reach 64.62%, 86.00%, and 81.36% for Z, PG, and ZPG, respectively. The CR removal% increased with increasing the initial pH of the CR solution for all adsorbents, reaching its maximum value at pH 7. The adsorption isotherms of CR onto Z, PG, and ZPG show that the Langmuir isotherm model is the best fit for Z, PG, and ZPG. The second-order diffusion model effectively handles CR adsorption onto Z, PG, and ZPG. Field tests revealed that the newly synthesized ZPG was effective in removing 82.1% dyes from industrial wastewater, confirming the foundation of a new eco-friendly nanoadsorbent that aid in the reuse of industrial wastewater. Future research is needed to investigate the impacts of dissolved salts on the adsorption behaviors of zeolite and ZPG composites, as well as the testing of more composites with different PG/Z ratios.

## Supplementary Information

Supplementary Information.
