# Supplementary material for: Design, characterization, and adsorption properties of Padina gymnospora/zeolite nanocomposite for Congo red dye removal from wastewater
Source: Sci Rep. 2021 Oct 26;11:21058. doi: 10.1038/s41598-021-00025-y (PMC8548541; doi:10.1038/s41598-021-00025-y)
Supplement: Supplementary file 1 — Supplementary Information. [file 41598_2021_25_MOESM1_ESM.docx]

**Design, characterization, and adsorption properties of Padina gymnospora/zeolite nanocomposite for Congo red dye removal from wastewater**

**Asmaa Ragab Dryaz^1^, Mohamed Shaban^2,3*^, Hamad AlMohamadi^4^, Khulood A. Abu Al-Ola^5^, Ahmed Hamd^3,6^, N.K. Soliman^6^, Sayed A. Ahmed^1^**

^1^ Department of Chemistry, Faculty of Science, Beni-Suef University, Beni Suef 62511, Egypt

^2^Department of Physics, Faculty of Science, Islamic University in Almadinah Almonawara, Almadinah Almonawara, 42351, Saudi Arabia.

^3^Nanophotonics and Applications Lab, Physics Department, Faculty of Science, Beni-Suef University, Beni Suef 62514, Egypt

^4^Department of Chemical Engineering, Faculty of Engineering, Islamic University of Madinah, Madinah, Saudi Arabia

^5^Department of Chemistry, College of Science, Taibah University, 30002 Al-Madinah Al-Munawarah, Saudi Arabia

^6^Basic Science Department, Nahda University Beni-Suef (NUB), Beni Suef, Egypt

***Corresponding author: mssfadel@aucegypt.edu**

1. **Adsorption isotherms**

Langmuir, Freundlich, and Tempkin isotherms have been applied to explain the reaction isotherm of the designed Z, PG and ZPG nanocomposite for the tested CR. The three models can be represented by equations 1, 2, and 3, respectively [1-4]:

$\frac{C_{e}}{q_{e}}= \frac{1}{K_{L}Q_{o}}+\frac{C_{e}}{Q_{o}}$ (1)

$\log q_{e}= \log K_{F}+\frac{1}{n}\log C_{e}$ (2)

$q_{e}= B ln K_{T}+B ln C_{e}$ (3)

Here, Q_o_ is the maximum amount of dye removed by Z, PG and ZPG adsorbents (mg/g); K_L_, K_F,_ and K_T_ indicate to Langmuir constant, Freundlich constant, and Tempkin binding constant, respectively. B(=RT/b) is a constant associated with the adsorbed heat, n is the adsorption density, T is the absolute temperature, and R is the universal gas constant.

1. **Adsorption kinetics and mechanism**

Different adsorption mechanisms and kinetics models such as intra-particle diffusion, pseudo-first-order,pseudo-second-order, and simple Elovich kinetic model are used for identifying the adsorption mechanisms and kinetics models that best match with the adsorption of CR onto Z, PG and ZPG adsorbents.

Equations 4 to 7 are used to represent the pseudo-first-order, pseudo-second-order, simple Elovich kinetic, and Intra-particle diffusion models, respectively [5-11].

ln (q_e_ – q_t_) = ln(q_e_) – k_1_ t (4)

$\frac{t}{q_{t}} = \frac{1}{k_{2}q_{e}^{2}}+\frac{t}{q_{e}}$ (5)

$q_{t} = \frac{1}{\beta}\ln\alpha\beta+\frac{1}{\beta}\ln t$ (6)

$q_{t} = k_{3}t^{\frac{1}{2}}+I$ (7)

Where k1, k2, and $k_{3}$represent rate constants of the pseudo-first-order, pseudo-second-order, and Intra-particle propagation models. *I* refers to a constant related to the boundary thickness. α implies the adsorption rate at time = 0 min(mg/min) and β represents the extent of surface coverage (g/mg).

Table S1. Conditions of experimental adsorption tests.

| **Series** | **Dye concentration, ppm** | **Z, PG and ZPG weight, g** | **Temperature, ^o^C** | **pH value** |
| --- | --- | --- | --- | --- |
| **1** | 5, 10, 15, 20 and 25 | 0.02 | 25 | 7 |
| **2** | 10 | 0.01, 0.02, 0.03, 0.04 and 0.05 | 25 | 7 |
| **3** | 10 | 0.02 | 25, 40, 50, 60,70, 80 and 90 | 7 |
| **4** | 10 | 0.02 | 25 | 3, 5, 7, and 10 |

Table S2. Characteristic wavenumbers and function groups of FTIR bands for Z, PG and ZPG adsorbents.

| **FT-IR peaks (cm^-1^)** | | | **Assignment** | **References** |
| --- | --- | --- | --- | --- |
| **Z** | **PG** | **ZPG** |  |  |
| - | 3624 | 3300 – 3500 | amine group (-NH) stretching | [12] |
| 3452, 3432, and 3442 | 3432 | 2925 and 2935 | hydroxyl group (-OH) | [12] |
| - | 2935 | 1425 | (-CH) group | [13, 14] |
| - | 1637 | 1019 | (-C=O) group | [15] |
| 1029 | - | 1030 | Si-O-Al | [16] |
| 464 | - | 460 | Si-O-Si bending | [17] |
| 400 - 800 | - | 400 - 800 | metal oxides | [18] |


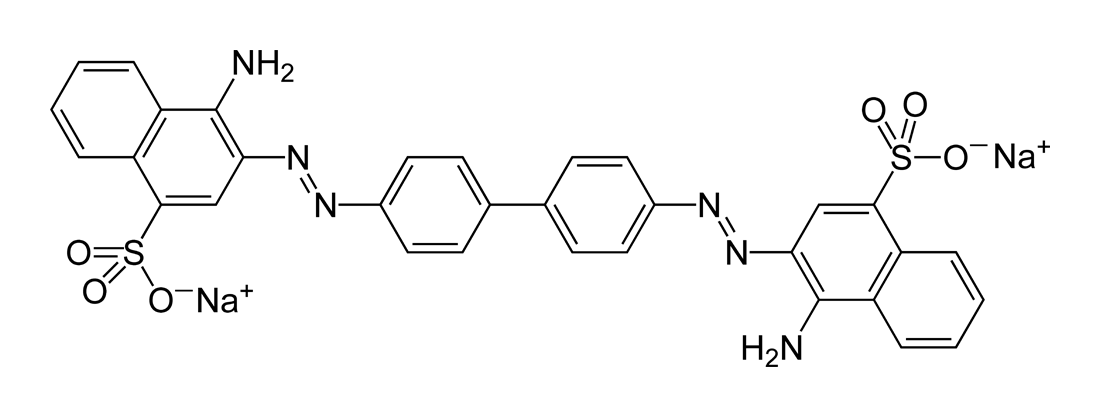


**Figure S1. Structure of Congo red**

**Figure S2. DLS spectra of Z, PG, and ZPG adsorbents.**


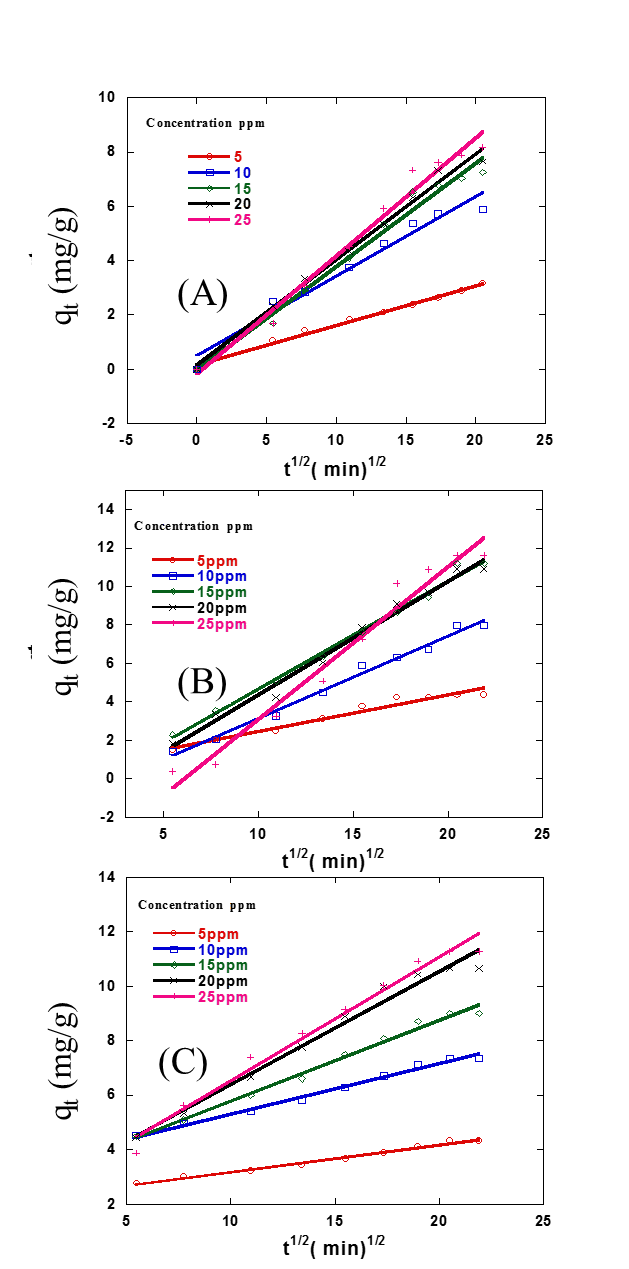


Figure S3. Intra-particle sorption kinetics of CR dye at 25 ^°^C and pH 7 by 20 mg of (A) Z, (B) PG, and (C) ZPG.


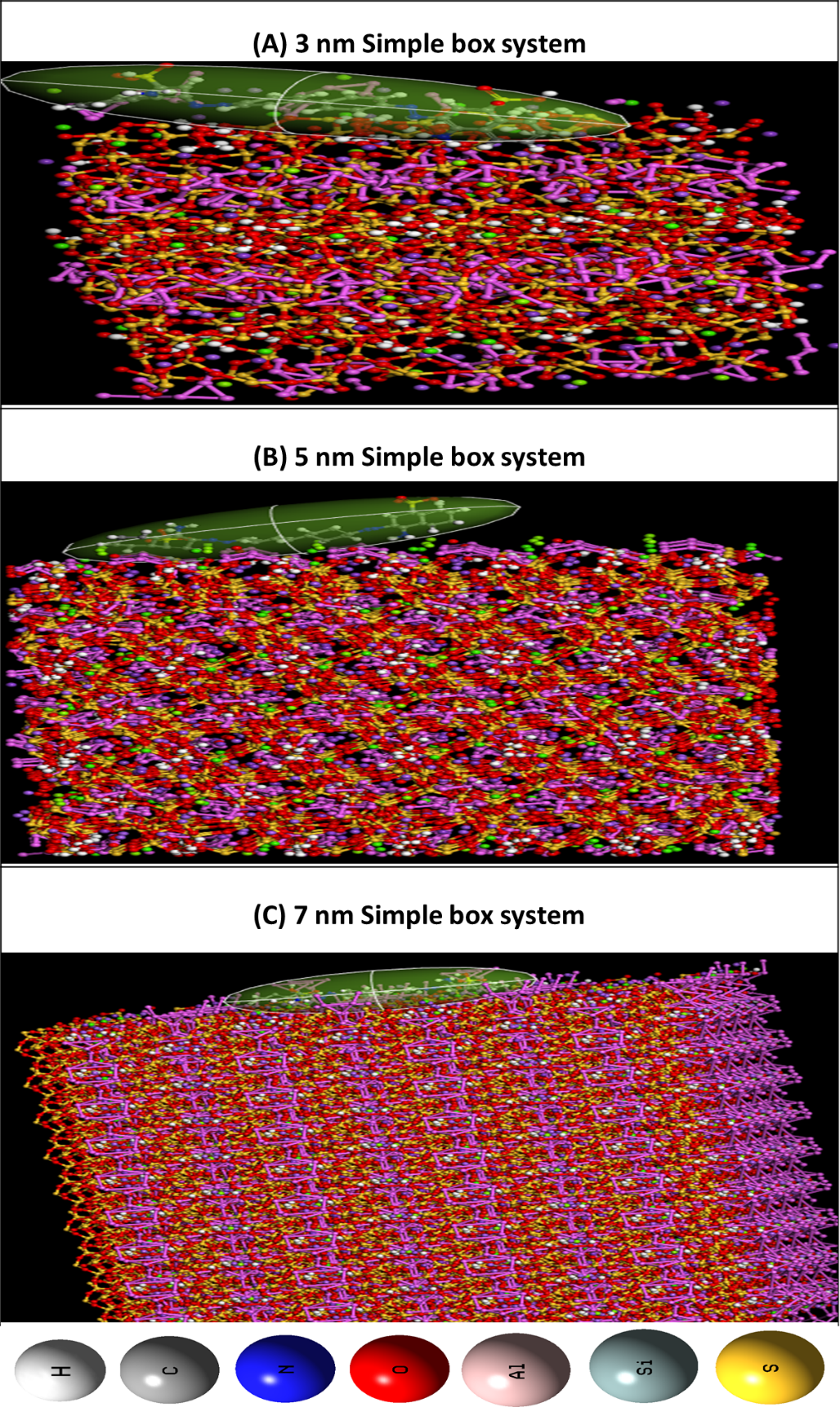


Figure S4. ***The adsorption configurations of adsorbed CR on zeolite clinoptilolite of 3, 5, and 7 nm simple box systems*** for clarity purpose.

**References**
[1] I. Langmuir, The adsorption of gases on plane surfaces of glass, mica and platinum, Journal of the American Chemical society 40(9) (1918) 1361-1403.

[2] H. Freundlich, Over the adsorption in solution, J. Phys. Chem 57(385471) (1906) 1100-1107.

[3] K. Foo, B.H. Hameed, Insights into the modeling of adsorption isotherm systems, Chemical engineering journal 156(1) (2010) 2-10.

[4] M. Temkin, V. Pyzhev, Kinetics of ammonia synthesis on promoted iron catalysts, Acta physiochim. URSS 12(3) (1940) 217-222.

[5] N. Soliman, H.S. Mohamed, R.H. Elsayed, N.M. Elmedny, A.H. Elghandour, S.A. Ahmed, Removal of chromium and cadmium ions from aqueous solution using residue of Rumex dentatus L. plant waste, Desalination and Water Treatment 149 (2019) 181-193.

[6] N. Xin, X. Gu, H. Wu, Y. Hu, Z. Yang, Application of genetic algorithm‐support vector regression (GA‐SVR) for quantitative analysis of herbal medicines, Journal of Chemometrics 26(7) (2012) 353-360.

[7] H.S. Mohamed, N. Soliman, D.A. Abdelrheem, A.A. Ramadan, A.H. Elghandour, S.A. Ahmed, Adsorption of Cd2+ and Cr3+ ions from aqueous solutions by using residue of Padina gymnospora waste as promising low-cost adsorbent, Heliyon 5(3) (2019) e01287.

[8] L. Fan, C. Luo, M. Sun, H. Qiu, X. Li, Synthesis of magnetic β-cyclodextrin–chitosan/graphene oxide as nanoadsorbent and its application in dye adsorption and removal, Colloids and Surfaces B: Biointerfaces 103 (2013) 601-607.

[9] H. Demiral, G. Gündüzoğlu, Removal of nitrate from aqueous solutions by activated carbon prepared from sugar beet bagasse, Bioresource technology 101(6) (2010) 1675-1680.

[10] N. Soliman, H.S. Mohamed, S.A. Ahmed, F.H. Sayed, A.H. Elghandour, S.A. Ahmed, Cd2+ and Cu2+ removal by the waste of the marine brown macroalga Hydroclathrus clathratus, Environmental Technology & Innovation (2019) 100365.

[11] F.-C. Wu, R.-L. Tseng, R.-S. Juang, Initial behavior of intraparticle diffusion model used in the description of adsorption kinetics, Chemical engineering journal 153(1) (2009) 1-8.

[12] W.M. Ibrahim, A.F. Hassan, Y.A. Azab, Biosorption of toxic heavy metals from aqueous solution by Ulva lactuca activated carbon, Egyptian journal of basic and applied sciences 3(3) (2016) 241-249.

[13] P. Nautiyal, K. Subramanian, M. Dastidar, Adsorptive removal of dye using biochar derived from residual algae after in-situ transesterification: alternate use of waste of biodiesel industry, Journal of environmental management 182 (2016) 187-197.

[14] M. Ruthiraan, E. Abdullah, N. Mubarak, M. Noraini, A promising route of magnetic based materials for removal of cadmium and methylene blue from waste water, Journal of environmental chemical engineering 5(2) (2017) 1447-1455.

[15] M.M. Ghoneim, H.S. El-Desoky, K.M. El-Moselhy, A. Amer, E.H.A. El-Naga, L.I. Mohamedein, A.E. Al-Prol, Removal of cadmium from aqueous solution using marine green algae, Ulva lactuca, The Egyptian Journal of Aquatic Research 40(3) 235-242.

[16] E. Horvath, J. Kristof, R.L. Frost, Vibrational Spectroscopy of Intercalated Kaolinites. Part I, Applied Spectroscopy Reviews 45(2) (2010) 130-147.

[17] J. Madejova, P. Komadel, Baseline studies of the clay minerals society source clays: infrared methods, Clays and clay minerals 49(5) (2001) 410-432.

[18] M.H. Masoudi R, Azin E, Taheri RA. , Adsorption of cadmium from aqueous solutions by novel Fe3O4-newly isolated Actinomucor sp. bio-nanoadsorbent: functional group study, Artificial cells, nanomedicine, and biotechnology 7 (2018) 1-10.
